# Novel Bragg peak FLASH radiotherapy: treatment of base-of-skull tumors within existing clinical guidelines

**DOI:** 10.3389/fonc.2026.1909184

**Published:** 2026-09-04

**Authors:** Michael Pennock, Balaji Selvaraj, Chingyun Cheng, Alex Kyler, Haibo Lin, Shaakir Hasan, Arpit Chhabra, Jehee Isabelle Choi, Richard Bakst, Rafi Kabarriti, Nancy Y Lee, Charles B. Simone, Minglei Kang

**Affiliations:** 1 Department of Radiation Oncology, University of Pittsburgh Hillman Cancer Center, Pittsburgh, PA, United States; 2 New York Proton Center, New York, NY, United States; 3 Department of Radiation Medicine, University of Wisconsin-Madison, Madison, WI, United States; 4 Department of Radiation Oncology, Montefiore Einstein Comprehensive Cancer Center, Bronx, NY, United States; 5 Icahn School of Medicine at Mount Sinai, New York, NY, United States; 6 Memorial Sloan Kettering Cancer Center, New York, NY, United States

**Keywords:** base of skull tumors, Bragg peak FLASH, FLASH radiotherapy, pencil beam scanning, proton

## Abstract

**Background and purpose:**

Proton therapy is commonly delivered for base-of-skull (BOS) tumors to minimize risk of normal-tissue injury. Novel Bragg peak FLASH may improve protection for organs at risk (OARs). We investigated if proton pencil-beam scanning (PBS) Bragg peak FLASH could achieve comparable dosimetry at ultra-high dose rates to conventional-rate (CONV)-intensity-modulated proton therapy (IMPT) for potential clinical use.

**Materials and methods:**

PBS Bragg peak FLASH and CONV-IMPT plans were optimized for 10 consecutive patients with BOS tumor in an in-house treatment-planning system and hypofractionation regimen (30 GyE in five fractions) to assess dosimetric quality while reflecting clinical practice, parameters, and hypothetical fraction doses for FLASH effect. FLASH dose-rate coverage (*V*_40Gy/s_) and dose-rate volume histograms (DRVHs) were quantified across OARs. FLASH (≥40 Gy/s) was achieved with a minimum MU per spot of 300–500 and a minimum spot time (MST) of 0.5 ms. FLASH dose-rate coverage, OAR sparing, and target coverage were averaged across all cases.

**Results:**

FLASH generated higher CTV *D*_max_ than CONV-IMPT (114% vs. 108%). Dose metrics for OARs were comparable between modalities (*p* > 0.05). Average dose rate (ADR) DRVHs of OARs indicated 74.1% *V*_40Gy/s_ FLASH dose-rate coverage without dose threshold, and 94.4% *V*_40Gy/s_ FLASH dose-rate coverage with 1-Gy dose threshold.

**Conclusion:**

PBS Bragg peak FLASH can deliver conformal target dosimetric coverage, ultra-high dose rates, and comparable OAR dosimetry to CONV-IMPT, suggesting that this novel technique is feasible for BOS tumor FLASH radiotherapy. The study supports future research to explore FLASH’s biological and clinical benefits in BOS tumors and OAR sparing.

## Highlights

- Efficacy of radiotherapy for BOS tumors is limited by the tolerance and proximity of nearby normal tissues.
- Bragg peak FLASH can deliver conformal target coverage and ultra-high dose rates.
- Bragg peak FLASH and CONV-IMPT achieve comparable OAR dose metrics and OAR sparing in the planning system.
- Bragg peak FLASH may maintain tumor control and enhance OAR sparing via the FLASH effect.

## 1 Introduction

Concurrent chemoradiotherapy (CRT) and surgery followed by radiotherapy (RT) are the standard of care in head and neck cancers (1, 2). RT in this region can be associated with substantial toxicity and morbidity (3). The skull base is anatomically complex, and base-of-skull (BOS) tumors are heterogeneous with varying aggressiveness (4, 5). RT is effective as first-line or adjuvant treatment, but can cause severe side effects, requiring new treatment approaches to improve therapeutic ratio (5).

Radiation dose to organs at risk (OARs) is dose-limiting when irradiating BOS tumors, and treatment efficacy may decrease if OAR preservation is prioritized. Despite the evolution of photon intensity-modulated RT (IMRT), undesirable doses are still deposited in OARs. Charged particle therapy has shown better clinical outcomes in comparison to conventional photon irradiation, with relatively few significant complications (6). Pencil-beam scanning (PBS) proton therapy (PT) depositing dose at a near-monoenergetic Bragg peak has enhanced conformality, dosimetry, and therapeutic ratio, maintaining comparable control to IMRT with less toxicity (7, 8). Studies, to date, have demonstrated excellent local control and low toxicity rates achieved with PBS for BOS tumors (9).

BOS radiation causes toxicity in a volume-dependent fashion (10, 11); BOS PT has been shown to improve conformality and OAR volumetric dose reduction over photon volumetric modulated arc therapy (VMAT) (12–15). However, proton *D*_max_ in the brain may be higher than that expected, increasing the risk of necrosis (16).

FLASH-RT (ultra-high dose rate > 40 Gy/s) is a groundbreaking modality that could possibly enable superior normal-tissue sparing and similar local control to conventional-rate (CONV) modalities in preclinical models (17). Murine studies have demonstrated tissue-sparing effects for proton FLASH (18, 19), and decreased toxicity has been associated with shortest delivery time and pulse number (20). In neurological tissues, CONV radiation causes neuroinflammation-mediated damage, and preclinical studies have shown that FLASH can preserve neuronal integrity, minimize normal-tissue toxicities, and possibly reduce the risk of cognitive deficit, although these data and the hypothesis that FLASH allows CNS dose escalation while better preserving neural tissue require cross-validation with functional outcomes (21, 22). Raster-scanning PBS FLASH at 120 Gy/s demonstrated reduced inflammation and preserved neurons compared to CONV radiation (19).

Research is ongoing to elucidate underlying FLASH radiobiology. Prior theories cited oxygen depletion, while current prevailing theories emphasize stem-cell preservation (23–25). FLASH demonstrated tumor control and minimal normal tissue damage in a human case (26). This potential increase in therapeutic ratio and conformality offered by FLASH has generated exploration into FLASH delivery via new or modified systems (27, 28).

Original studies of proton FLASH, including the first FLASH clinical trial (29, 30), have required PBS transmission FLASH beams to achieve ultra-high dose rates, which traverse the entire patient and deliver significant doses to critical structures (31). It is still desirable to reduce dose as much as possible to normal tissues, even if FLASH can be delivered. PBS Bragg peak FLASH, a developing technology, has almost zero exit dose, but the extent of its dosimetric performance remains undefined. A modifying factor uniting modality, energy, dose, fractionation, tissue types, and early and late effects has yet to be defined. By combining modifications to the range shifter, range compensator (RC), spot map, and inverse planning system (32), we propose that PBS Bragg peak FLASH is capable of conformality, OAR sparing, and adequate FLASH dose-rate coverage for BOS tumors.

This proof-of-principle work evaluated novel PBS Bragg peak FLASH’s dosimetric potential and compared it to that of PBS CONV-intensity-modulated proton therapy (IMPT) in a cohort of patients with BOS tumor for target coverage and OAR sparing. FLASH treatment plans were evaluated for OAR sparing and the ability to achieve sufficiently high FLASH dose-rate coverage with realistic dose thresholds under multiple-field optimization. We predict that PBS Bragg peak FLASH plans can achieve ultra-high dose rates, deliver similar OAR dose metrics and target coverage to CONV-IMPT, and align with current clinical proton delivery.

## 2 Materials and methods

A novel FLASH delivery technique was developed that employs a universal range shifter (URS) and a beam-specific RC to retract the proton range of the cyclotron’s highest single-energy beam, enabling highly conformal FLASH dose delivery (32, 33). This FLASH technique was implemented to matRad, an open-source toolkit enabling dosimetric accuracy in three-dimensional intensity-modulated radiation therapy planning for scanned protons and carbon ions (34). For each patient, BP-FLASH plans were generated using the same beam arrangement and prescription as the corresponding conventional IMPT plans to enable a direct dosimetric comparison. Treatment planning was initiated with inverse optimization to satisfy target coverage and OAR dose objectives under the single-energy delivery configuration. The optimized spot map was then iteratively refined by applying delivery-specific constraints, including the minimum monitor units (MMU) per spot and spot delivery time, to improve FLASH dose-rate coverage while maintaining clinically acceptable plan quality. To account for treatment uncertainties, a 3-mm setup uncertainty and a 3.5% Hounsfield unit (HU)-to-relative stopping power (RSP) uncertainty were incorporated into the RC design using a smearing approach (33). Following each optimization cycle, the plan was recalculated with the URS and patient-specific RCs incorporated into the dose calculation until both dosimetric objectives and FLASH delivery requirements were satisfied. This integrated planning workflow enables highly conformal Bragg peak dose distributions while remaining compatible with ultra-high dose-rate delivery.

The dose was reported in “GyE” units to account for a proton RBE of 1.1 and to show that RT was delivered in bio-equivalent doses to well-known photon RT prescriptions and OAR constraints. Similar target coverage goals and dose constraints were utilized during optimization to facilitate equal comparisons between the treatment methods. This method has been previously described (35, 36). All data were analyzed to identify data trends, generate dose distributions, and calculate dose-volume histograms (DVHs) for OAR sparing and target coverage of all FLASH and CONV-IMPT plans, and dose-rate volume histograms (DRVHs) for FLASH plans. Per ESTRO-ACROP consensus guidelines, updated central nervous system OAR constraints have been developed and were adapted for the current study (37).

The Varian ProBeam system (Varian Medical Systems, Palo Alto, CA, USA) was modeled in this platform with a 0.5-ms delivery time for the MMU per spot (38), defined as the minimum spot time (MST). Under FLASH settings, the spot-scanning speed was set to 10 mm per ms (36). In this configuration, the MST is fixed to deliver the MMU per spot in FLASH mode, while the proton beam current is adjusted accordingly to achieve this delivery. A larger MMU per spot requires a higher beam current to maintain FLASH dose-rate conditions (39). The treatment planning will automatically fine-tune the MMU to balance the plan quality and FLASH dose ratio for critical OARs (38, 40).

A sharp lateral penumbra is critical for achieving high-quality treatment in BOS cases, where numerous OARs are adjacent to the target. Although the system produced a small spot size of 3.3 mm (σ) at the highest energy of 250 MeV (41), the potential spot broadening caused by multiple Coulomb scattering (MCS) through the URS and RCs was carefully considered. To mitigate this effect, the range-pullback devices were positioned as close to the patient as possible during treatment planning to minimize air gaps and preserve a sharp lateral penumbra.

**Figure 1:**
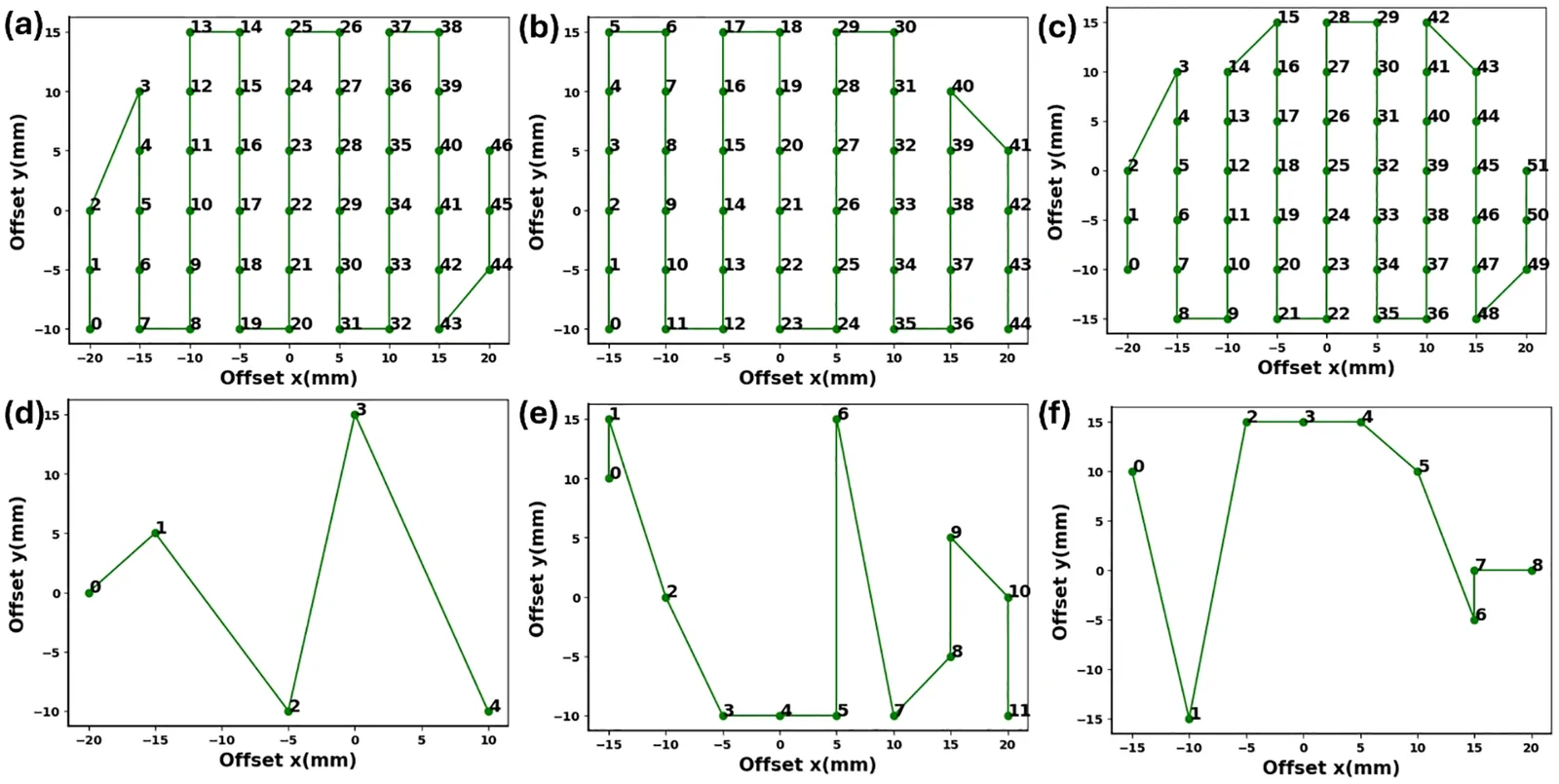
**(a–c)** FLASH Bragg peak plans were optimized with a small MMU per spot to ensure plan quality (upper spot maps). Low-weight spots were removed to improve dose rate. Finally, we continued to optimize the plans with a higher minimum MU per spot constraint **(d–f)** (sparse spot maps).

FLASH dose-rate coverage was quantified using the volume receiving a dose rate ≥40 Gy/s (*V*_40Gy/s_), as previously introduced (38). FLASH dose-rate coverage was evaluated on a field-by-field basis using the previously published proton PBS average dose rate (ADR) method, which accounts for both spot delivery and scanning dynamics. The mathematical definitions and calculation framework for both ADR and *V*_40Gy/s_ have been described previously (36, 38, 40). In the present study, the FLASH coverage metric *V*_40Gy/s_ was defined as the fraction of voxels within the region of interest (ROI) that received both a dose greater than or equal to a prescribed dose threshold (d^*^) and a dose rate of at least 40 Gy/s, as expressed in Equation 1:

(1) $$V_{40Gy/s}=\frac{\sum_{j=1}^{M}I\left(D_{j}\geq d^{*},{\dot{D}}_{j}\geq 40Gy/s\right)}{\sum_{j=1}^{M}I\left(D_{j}\geq d^{*}\right)}$$

where *D_j_* and ${\dot{D}}_{j}$ denote the dose and dose rate of voxel *j*, respectively, *M* is the number of voxels within the ROI, and *I*(·) is an indicator function equal to 1 when the specified condition is satisfied and 0 otherwise. The dose threshold d^*^ was set to 0 Gy (default), 1 Gy, and 5 Gy, to assess the FLASH dose-rate coverage under different dose thresholds. A spot-map optimization tool accounted for dose rate, spot times, plan quality, and MMU constraints in inverse planning and allowed conformal, IMPT-equivalent dosimetry via inverse multiple-field optimization (38, 40). The novel spot-map optimization approach significantly reduces the total number of spots by removing low-weight spots, thereby enabling ultra-fast delivery while maintaining high-quality dosimetry. As shown in **Figures 1a–c**, which displays spot maps for three representative fields, the optimized maps exhibit a marked reduction in spot density, yielding a highly sparse distribution that supports FLASH-capable delivery, as illustrated in **Figures 1d–f**.

After IRB approval, PBS Bragg peak FLASH (≥40 Gy/s) and CONV-IMPT plans were created, optimized, and compared retrospectively for 10 consecutive patients to receive the same dose and fractionation. Per research showing FLASH tissue-sparing starts at 40 Gy/s and 5–10 Gy/fraction (40), and the need to assess clinically relevant doses that are feasible with current FLASH-PBS technology, plans were optimized for 30 GyE in five fractions and compared across CONV-IMPT and PBS Bragg peak FLASH in each patient. Target coverage was normalized to 100% CTV receiving at least 95% prescribed dose. As the dose rate in the near-Bragg peak region can be ~40% lower than in the plateau region and worsened by an air gap, we utilized a high MMU per spot via a high nozzle current (38). FLASH plans were generated using the same beam arrangement as the CONV-IMPT plans to minimize bias in plan comparison (32). Two-tailed paired *t*-tests were used to analyze the statistical differences in dose metrics between CONV-IMPT and BP-FLASH plans. *p* < 0.05 was considered statistically significant.

## 3 Results

**Figure 2:**
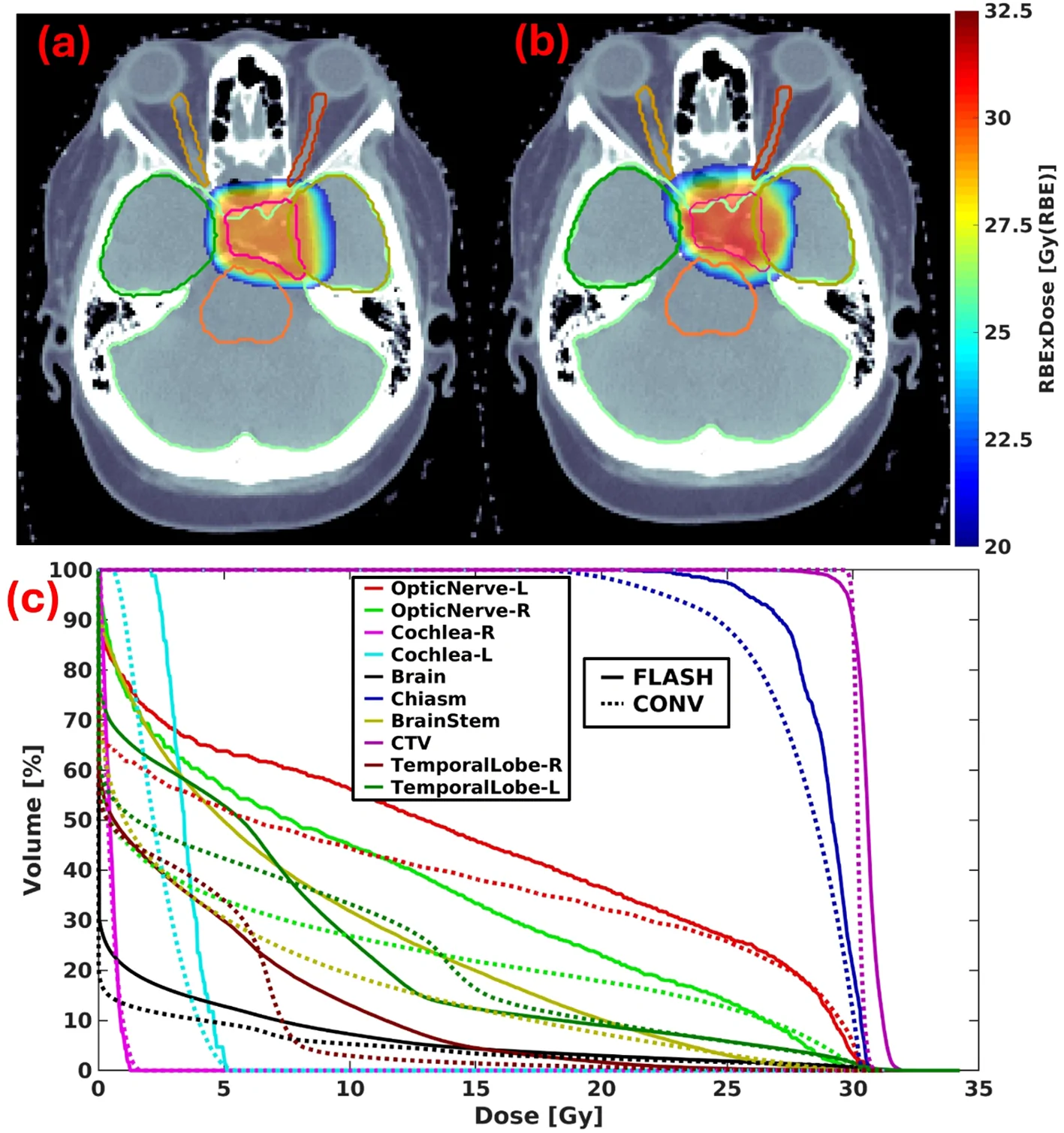
The dosimetric comparison between CONV-IMPT and BP-FLASH plans for a representative patient. **(a)** 2D dose distribution of CONV-IMPT. **(b)** 2D dose distribution of BP-FLASH. **(c)** DVH comparing dose coverage across all structures for CONV-IMPT and BP-FLASH.

All 10 patients with BOS tumor were averaged to investigate target and OAR statistics. Dose metrics for most OARs, including temporal lobe, spinal cord, brain, brainstem, cochlea, optic chiasm, and optic nerves, were comparable and not significantly different between PBS Bragg peak FLASH and CONV-IMPT (*p* > 0.05), but for optic chiasm, CTV *D*_max_, brainstem, and brain *D*_max_, Bragg peak FLASH deposited significantly more dose (**Table 1**; **Figure 2**). The 2D dose distribution for one representative patient treated with 30 GyE in five fractions is depicted in **Figure 2**.

**Table 1:** Dose metrics averaged across all 10 patients for CTV coverage and normal tissue doses between PBS Bragg peak FLASH and CONV-IMPT.

Target and OARs	Metric [ideal constraint, percent (%) or equivalent centigray (cGyE)]	IMPT mean, percent (%) or equivalent centigray (cGyE) (standard deviation)	Bragg peak FLASH mean, percent (%) or equivalent centigray (cGyE) (standard deviation)	*p*-value	Difference (%)
TemporalLobe_L	*D*_mean_ (<1,500 cGy)	539.5 (± 135.4)	583.5 (± 141.3)	0.135	1.5
TemporalLobe_R	*D*_mean_ (<2,000 cGy)	351.4 (± 100.4)	430.5 (± 152.1)	0.073	2.6
SpinalCord	*D*_max_ (<2,300 cGy)	85.9 (± 64.0)	191.3 (± 90.9)	0.096	3.5
Parotid_R	*D*_mean_ (<1,500 cGy)	401.3 (± 179.4)	656.5 (± 293.6)	0.500	8.5
Parotid_L	*D*_mean_ (<1,500 cGy)	0.1 (± 0.02)	2.7 (± 1.2)	0.500	0.1
OpticNerve_L	*D*_max_ (<2,500 cGy)	2,060.4 (± 432.2)	2,265.6 (± 441.8)	0.057	6.8
OpticNerve_R	*D*_max_ (<2,500 cGy)	2,011.2 (± 407.4)	2,210.4 (± 374.1)	0.179	6.6
Cochlea_L	*D*_max_ (<2,300 cGy)	667.6 (± 293.9)	899.6 (± 273.9)	0.051	7.7
Cochlea_R	*D*_max_ (<2,300 cGy)	897.1 (± 343.4)	1,084.5 (± 381.5)	0.077	6.2
Chiasm	*D*_max_ (<2,500 cGy)	2,213.2 (± 416.0)	2,478.5 (± 390.0)	0.005*	8.8
CTV	*D*_95%_ (>95%)	92.3 (± 4.0)	93.9 (± 2.7)	0.285	1.5
	*D*_max_ (<115%)	107.3 (± 0.9)	114.4 (± 1.4)	<0.001*	7.1
	*V*_95%_ (>95%)	97.9 (± 1.9)	97.8 (± 2.1)	0.285	0.1
BrainStem	*D*_max_ (<3,000 cGy)	2,633.6 (± 241.6)	2,794.4 (± 243.7)	0.097	5.4
	*D*_0.5cm3_ (<2,000 cGy)	1,662.6 (± 318.1)	1,863.9 (± 312.6)	0.050*	6.7
Brain	*D*_max_ (<100%)	106.9 (± 0.8)	114.0 (± 1.7)	<0.001*	7

* Statistical significance.

**Figure 3:**
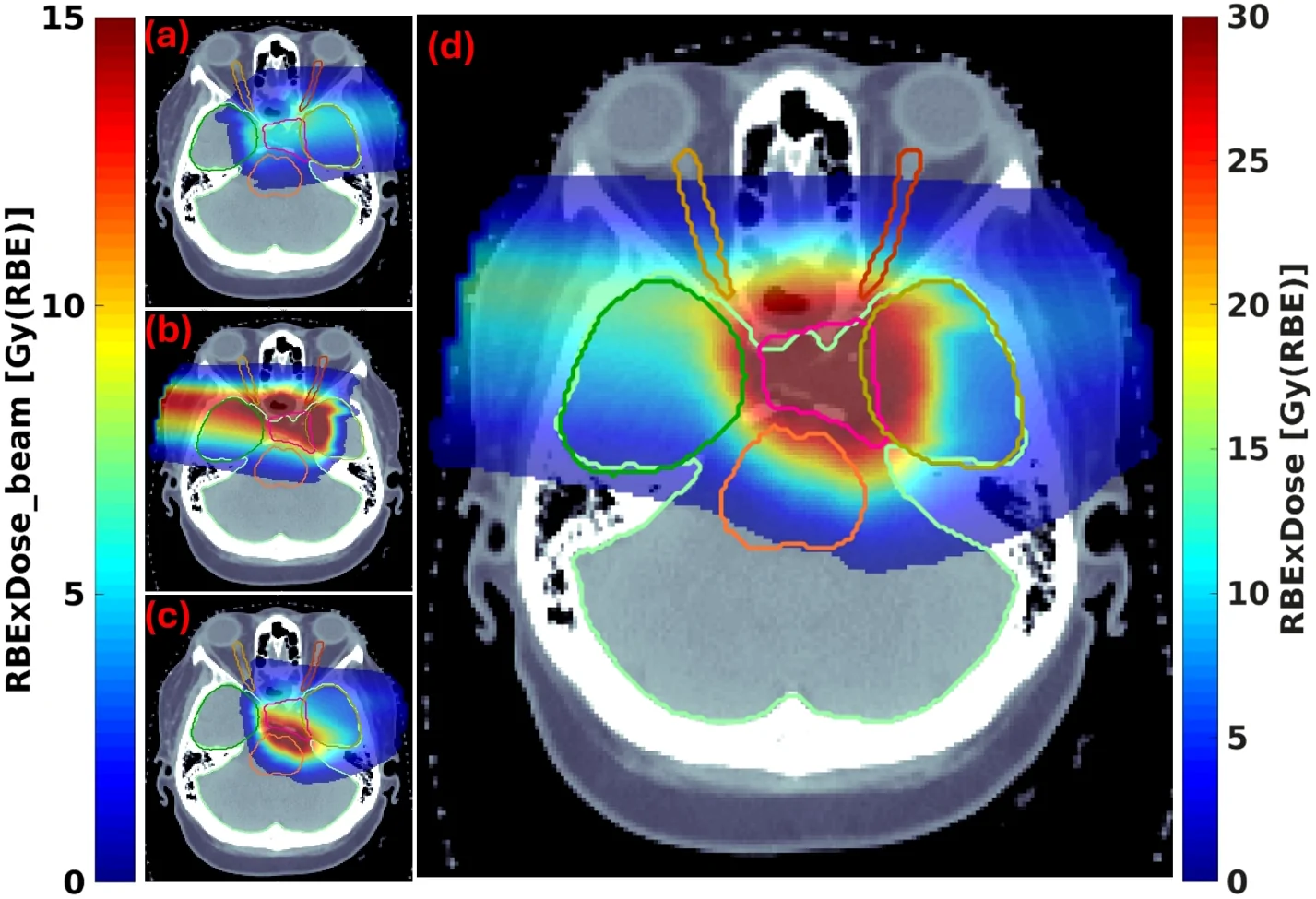
2D dose distribution for each beam angle and for beam-angle combination. **(a)** Beam 1 (beam angle 85, couch angle = 0). **(b)** Beam 2 (beam angle 275, couch angle = 0). **(c)** Beam 3 (beam angle 90, couch angle = 340). **(d)** Total dose.

**Figure 4:**
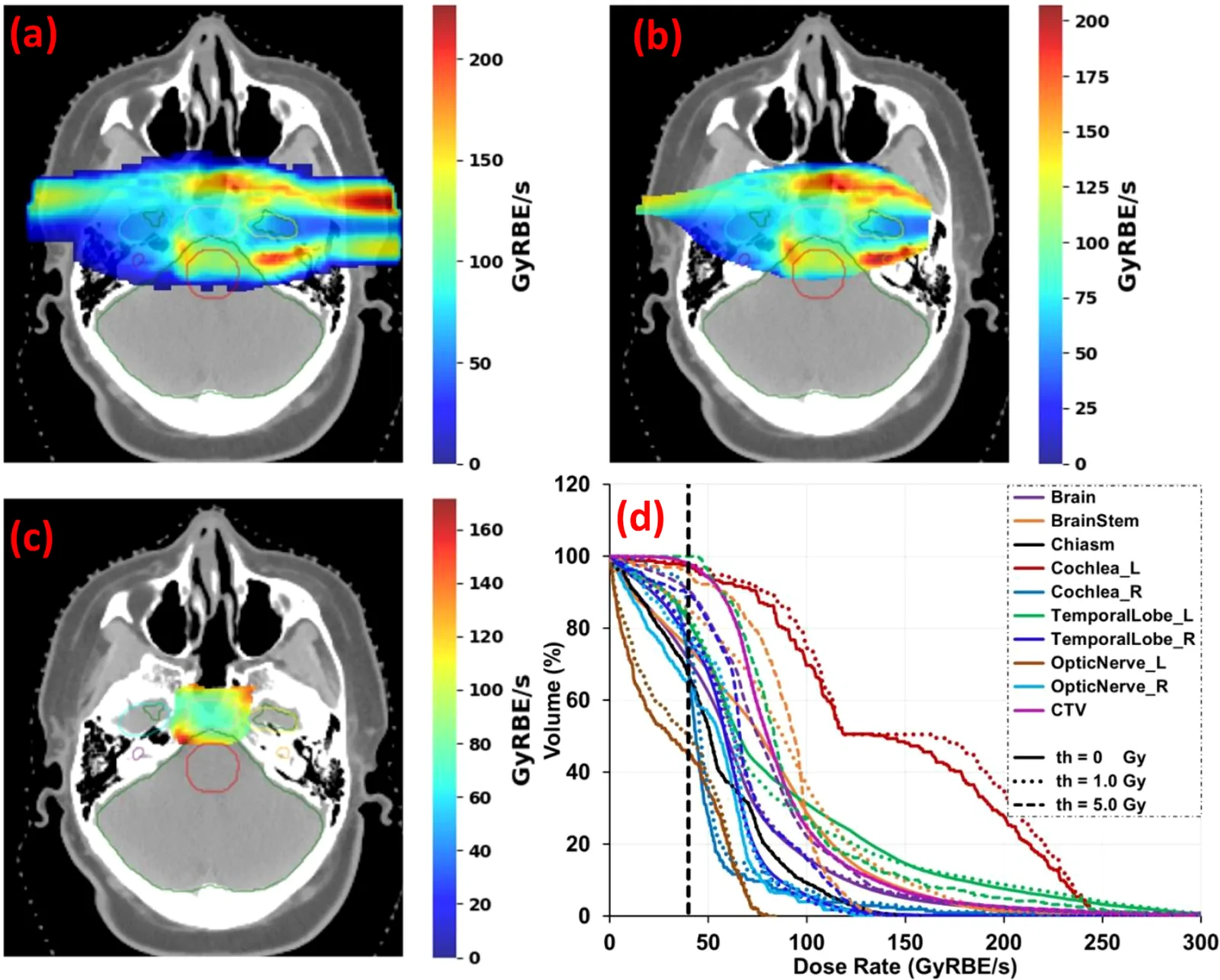
This is the 2D dose-rate distribution for a representative patient when different dose thresholds are applied to evaluate the FLASH ratio. As dose threshold increased from **(a)** no threshold GyE to **(b)** 1 GyE and **(c)** 5 GyE, volumetric FLASH dose-rate coverage increased in **(d)** (dose threshold = th).

**Figure 5:**
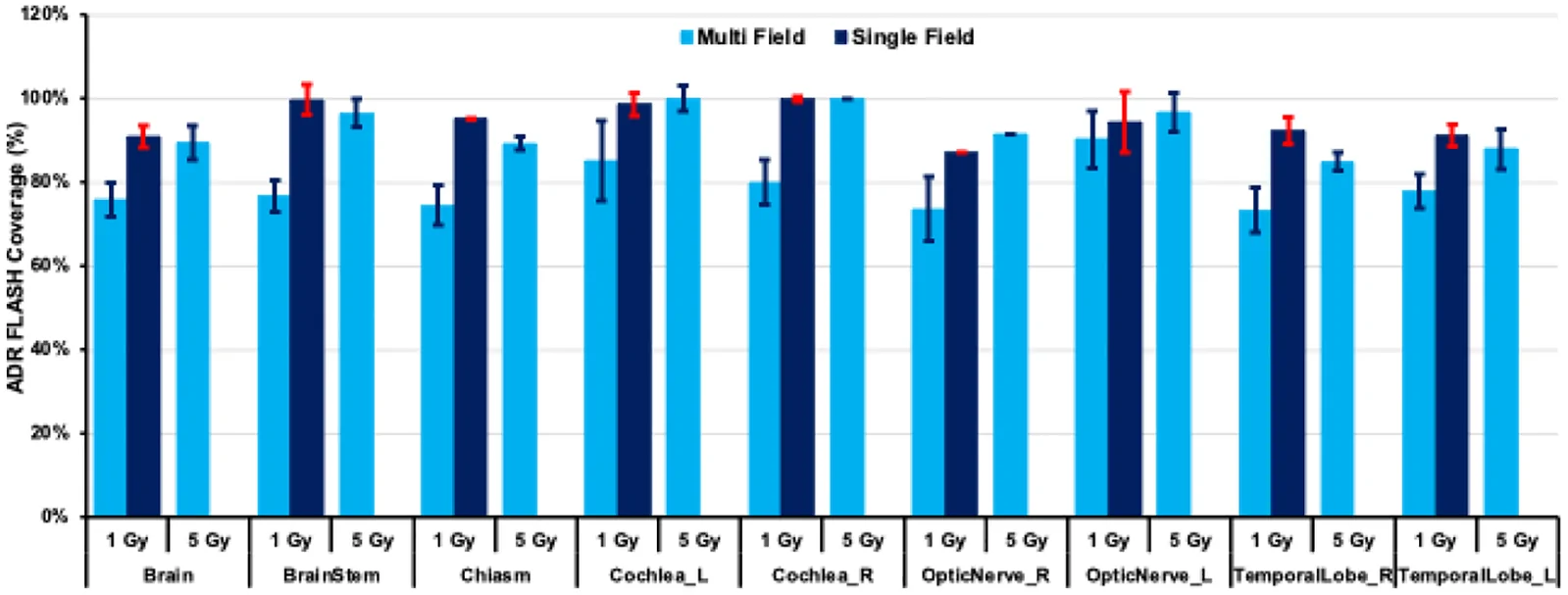
This bar graph shows the average dose rate (ADR) FLASH coverage for each OAR when different dose thresholds are applied (1 and 5 GyE), and how ADR differs within a dose threshold by single-field or multi-field delivery. ADR FLASH coverage (averaged across 10 patients) increased with dose threshold. ADR FLASH coverage increased with dose per field, and thus was higher for single-field plans than multi-field plans.

PBS Bragg peak FLASH doses for OARs were slightly higher than CONV-IMPT doses (all *p*-values > 0.05 except for chiasm, brain, and brainstem) (**Table 1**; **Figure 2**). The FLASH effect was more likely to occur when a single field delivered a dose above a dose threshold (**Figures 3**–**5**). A single-field approach is more conservative, as it evaluates FLASH effect for each beam individually (**Figure 3**). When higher dose thresholds were applied, higher FLASH ratios were demonstrated, and each fraction delivered 6 GyE using three to five beam angles (**Figures 4**, **5**). A single field could deliver a maximum dose of 3–4 GyE (no single field could achieve a 5-Gy dose threshold) (**Figure 5**). As dose and dose threshold per field increased, FLASH dose-rate coverage increased (**Figures 4**, **5**). DRVHs indicated 74.1% *V*_40Gy/s_ FLASH dose-rate coverage without a dose threshold and 94.4% *V*_40Gy/s_ FLASH dose-rate coverage with 1-Gy dose threshold applied in individual fields (**Figures 4**, **5**).

As dose threshold increased from 1 to 5 GyE, ADR FLASH coverage increased, dose-volume coverage became more conformal, and low-dose bath decreased (**Figures 4**, **5**). The less fields utilized, the greater the dose per field. When evaluating coverage on a per-field basis, assuming the FLASH effect occurs in each individual field, increased ADR FLASH coverage can be attained compared to the dose threshold applied in multi-field methods (**Figure 5**).

## 4 Discussion

This study demonstrates the dosimetric feasibility of the proposed single-energy PBS Bragg peak FLASH technique for BOS tumors. Compared with conventional IMPT, Bragg peak FLASH achieved comparable target coverage and OAR dose metrics while maintaining ultra-high dose-rate coverage *V*_40Gy/s_ (>90%) and eliminating energy switching through single-energy delivery. Although modest increases in CTV hotspot, brain, brainstem, and chiasm dose were observed, all treatment plans remained within clinically acceptable planning objectives. These findings indicate that the proposed Bragg peak FLASH planning approach can preserve clinically acceptable dosimetric quality while enabling FLASH-capable delivery. However, the increased hotspot and reduced target dose uniformity illustrate the inherent trade-off between maximizing FLASH dose-rate coverage and maintaining optimal dose conformity. The simplified single-energy delivery and reduced beam delivery time further support the investigation of this technique for future preclinical and clinical FLASH studies.

While the present study demonstrates the dosimetric feasibility of PBS Bragg peak FLASH for BOS tumors, the potential biological benefit of this technique remains uncertain. Current evidence suggests that the FLASH effect depends on multiple factors beyond achieving ultra-high dose rates, including total dose, dose per fraction, irradiation time, tissue type, oxygenation status, and the intrinsic radiobiological characteristics of individual organs (42, 43). Although numerous preclinical studies have reported encouraging normal tissue sparing, the underlying biological mechanisms remain incompletely understood, and there is currently insufficient experimental evidence to establish validated biological models capable of predicting tissue-specific FLASH responses, particularly for the central nervous system structures relevant to BOS treatment (44, 45). Consequently, the comparable OAR dosimetry and high FLASH dose-rate coverage achieved in this study should be interpreted as demonstrating the technical feasibility of Bragg peak FLASH planning rather than evidence of improved biological OAR protection. Future preclinical studies, biological modeling, and clinical investigations are needed to determine whether these favorable dosimetric characteristics translate into meaningful reductions in treatment-related toxicity and improved clinical outcomes for patients with BOS tumors (44, 45).

From a technical perspective, the single-energy Bragg peak delivery strategy introduces several dosimetric trade-offs that should also be considered. The Bragg peak at its highest energy resulted in a lack of freedom in energy modulation and reduced target uniformity from energy straggling, stochastic loss, and larger dose falloff, which was observed at low dose thresholds in the current study’s CTV coverage (40). The spot size for FLASH is larger than for CONV-IMPT from MCS, increasing low-dose spillage. The FLASH Bragg peak utilizes far fewer spots than CONV-IMPT, which compromises target dose uniformity. Despite these concerns, the current study’s treatment plans easily met accepted clinical dose constraints. Nevertheless, the observed increase in low-dose spillage and reduction in target dose uniformity may represent important dosimetric trade-offs that warrant consideration in clinical implementation.

This proof-of-principle study has several limitations. First, the dosimetric analysis was performed in a cohort of 10 consecutive patients with BOS tumor. Treatment planning feasibility studies are designed to evaluate dosimetric characteristics rather than clinical outcomes and therefore typically involve a relatively small number of representative patient datasets. Future studies including larger patient cohorts are warranted to further validate the robustness and generalizability of the proposed Bragg peak FLASH planning approach. We introduced the *V*_40Gy/s_ metric to describe FLASH dose-rate coverage. FLASH’s ultra-fast delivery should mitigate uncertainty from intra-beam motion, yet the current study did not account for uncertainty from inter-beam motion or gantry rotation. This study also did not explore range uncertainty robustness in BOS treatment planning (40), and future studies can investigate modifications to line scanning, dead times, overcoming MU saturation, and quantifying robustness via fluence maps, spot scanning, or group sparsity (35). OAR dose thresholds in FLASH can be challenging per zero exit dose, and planning goals may have to be optimized to FLASH dose thresholds rather than dose constraints. Future studies are indicated for spot and path optimization in relation to air gap, collimator, or aperture locations, and whether our QA, Monte Carlo simulation, LET predictions, and raster scanning are realistic in patient scenarios (19). Future studies can also investigate or cross-validate how FLASH-mediated biological changes can be stratified by dose-rate response (19, 21).

Another limitation of this study is that, although setup and range uncertainties were incorporated into the URS and RC design using a smearing approach, a dedicated robustness evaluation examining the combined effects of these uncertainties and reduced spot density was not performed. To achieve ultra-high dose-rate delivery, the Bragg peak FLASH plans employed fewer spots than conventional IMPT. Although a reduced spot density may increase sensitivity to treatment uncertainties, the single-energy Bragg peak FLASH technique also produces a broadened Bragg peak and enlarged lateral spot size, resulting in smoother dose gradients that may reduce sensitivity to setup and range perturbations. Our previous work demonstrated favorable robustness characteristics of this delivery approach under clinically relevant uncertainty scenarios (33). Nevertheless, future studies should perform comprehensive robustness analyses to evaluate the combined effects of setup uncertainty, range uncertainty, and reduced spot density on dose distribution and plan quality.

## 5 Conclusion

PBS Bragg peak FLASH achieved conformal target coverage, ultra-high dose-rate delivery, and OAR dosimetry that were largely comparable to conventional IMPT for BOS tumors. These findings demonstrate the dosimetric feasibility of this single-energy Bragg peak FLASH approach within current clinical planning objectives while highlighting the trade-offs between maximizing FLASH dose-rate coverage and maintaining optimal dose conformity. This proof-of-principle study supports further preclinical and clinical investigation to evaluate the biological effects and potential clinical benefits of BOS Bragg peak FLASH radiotherapy.

## Data Availability

The raw data supporting the conclusions of this article will be made available by the authors, without undue reservation.

## References

[1] Pignon J Bourhis J Domenge CO Designé L . Chemotherapy added to locoregional treatment for head and neck squamous-cell carcinoma: three meta-analyses of updated individual data. Lancet. (2000) 355:949–55. doi: 10.1016/S0140-6736(00)90011-4 10768432

[2] Petit C Pignon J Landais C Trotti A Gregoire V Overgaard J . What is the most effective treatment for head and neck squamous cell carcinoma?: An individual patient data network meta-analysis from the MACH-NC and MARCH collaborative groups. Eur J Cancer. (2017), S101–2. doi: 10.1016/s0959-8049(17)30415-x

[3] Nguyen-Tan PF Zhang Q Ang KK Weber RS Rosenthal DI Soulieres D . Randomized phase III trial to test accelerated versus standard fractionation in combination with concurrent cisplatin for head and neck carcinomas in the Radiation Therapy Oncology Group 0129 trial: long-term report of efficacy and toxicity. J Clin Oncol. (2014) 32:3858–67. doi: 10.1200/JCO.2014.55.3925 25366680 PMC4239304

[4] Goldbrunner R Stavrinou P Jenkinson MD Sahm F Mawrin C Weber DC . EANO guideline on the diagnosis and management of meningiomas. Neuro Oncol. (2021) 23:1821–34. doi: 10.1093/neuonc/noab150 34181733 PMC8563316

[5] Stacchiotti S Sommer J Chordoma Global Consensus G . Building a global consensus approach to chordoma: a position paper from the medical and patient community. Lancet Oncol. (2015) 16:e71–83. doi: 10.1016/S1470-2045(14)71190-8 25638683

[6] Chhabra AM Rice SR Holtzman A Choi JI Hasan S Press RH . Clinical outcomes and toxicities of 100 patients treated with proton therapy for chordoma on the proton collaborative group prospective registry. Radiother Oncol. (2023) 183:109551. doi: 10.1016/j.radonc.2023.109551 36813169

[7] Baumann BC Mitra N Harton JG Xiao Y Wojcieszynski AP Gabriel PE . Comparative effectiveness of proton vs photon therapy as part of concurrent chemoradiotherapy for locally advanced cancer. JAMA Oncol. (2020) 6:237–46. doi: 10.1001/jamaoncol.2019.4889 31876914 PMC6990870

[8] Amichetti M Amelio D Minniti G . Radiosurgery with photons or protons for benign and Malignant tumours of the skull base: a review. Radiat Oncol. (2012) 7:210. doi: 10.1186/1748-717X-7-210 23241206 PMC3552759

[9] Lesueur P Calugaru V Nauraye C Stefan D Cao K Emery E . Proton therapy for treatment of intracranial benign tumors in adults: A systematic review. Cancer Treat Rev. (2019) 72:56–64. doi: 10.1016/j.ctrv.2018.11.004 30530009

[10] Martin JD Buckley AR Graeb D Walman B Salvian A Hay JH . Carotid artery stenosis in asymptomatic patients who have received unilateral head-and-neck irradiation. Int J Radiat Oncol Biol Phys. (2005) 63:1197–205. doi: 10.1016/j.ijrobp.2005.04.017 15978738

[11] Wen DW Lin L Mao YP Chen CY Chen FP Wu CF . Normal tissue complication probability (NTCP) models for predicting temporal lobe injury after intensity-modulated radiotherapy in nasopharyngeal carcinoma: A large registry-based retrospective study from China. Radiother Oncol. (2021) 157:99–105. doi: 10.1016/j.radonc.2021.01.008 33484752

[12] Simone CB Ly D Dan TD Ondos J Ning H Belard A . Comparison of intensity-modulated radiotherapy, adaptive radiotherapy, proton radiotherapy, and adaptive proton radiotherapy for treatment of locally advanced head and neck cancer. Radiother Oncol. (2011) 101:376–82. doi: 10.1016/j.radonc.2011.05.028 21663988 PMC3174314

[13] Dutz A Luhr A Troost EGC Agolli L Butof R Valentini C . Identification of patient benefit from proton beam therapy in brain tumour patients based on dosimetric and NTCP analyses. Radiother Oncol. (2021) 160:69–77. doi: 10.1016/j.radonc.2021.04.008 33872640

[14] Zhang Y Huo W Adams J Sanford N Lam M Lu Y . Temporal lobe necrosis after proton for nasopharyngeal carcinoma: Predictive factors and clinical RBE estimation. Int J Radiat Oncol Biol Phys. (2017) 99:E386. doi: 10.1016/j.ijrobp.2017.06.152638826717

[15] Adeberg S Harrabi SB Bougatf N Verma V Windisch P Bernhardt D . Dosimetric comparison of proton radiation therapy, volumetric modulated arc therapy, and three-dimensional conformal radiotherapy based on intracranial tumor location. Cancers (Basel). (2018) 10:401. doi: 10.3390/cancers10110401 30373115 PMC6266019

[16] Garbacz M Cordoni FG Durante M Gajewski J Kisielewicz K Krah N . Study of relationship between dose, LET and the risk of brain necrosis after proton therapy for skull base tumors. Radiother Oncol. (2021) 163:143–9. doi: 10.1016/j.radonc.2021.08.015 34461183

[17] Montay-Gruel P Petersson K Jaccard M Boivin G Germond J-F Petit B . Irradiation in a flash: Unique sparing of memory in mice after whole brain irradiation with dose rates above 100 Gy/s. Radiother Oncol. (2017) 124:365–9. doi: 10.1016/j.radonc.2017.05.003 28545957

[18] Kim MM Verginadis II Goia D Haertter A Shoniyozov K Zou W . Comparison of FLASH proton entrance and the spread-out Bragg peak dose regions in the sparing of mouse intestinal crypts and in a pancreatic tumor model. Cancers (Basel). (2021) 13:4244. doi: 10.3390/cancers13164244 34439398 PMC8392865

[19] Dokic I Meister S Bojcevski J Tessonnier T Walsh D Knoll M . Neuroprotective effects of ultra-high dose rate FLASH Bragg peak proton irradiation. Int J Radiat Oncol Biol Phys. (2022) 113:614–23. doi: 10.1016/j.ijrobp.2022.02.020 35196536 PMC11034835

[20] Pratx G Kapp DS . A computational model of radiolytic oxygen depletion during FLASH irradiation and its effect on the oxygen enhancement ratio. Phys Med Biol. (2019) 64:185005. doi: 10.1088/1361-6560/ab3769 31365907

[21] Montay-Gruel P Markarian M Allen BD Baddour JD Giedzinski E Jorge PG . Ultra-high-dose-rate FLASH irradiation limits reactive gliosis in the brain. Radiat Res. (2020) 194:636–45. doi: 10.1667/RADE-20-00067.1 32853387 PMC7856066

[22] Montay-Gruel P Acharya MM Petersson K Alikhani L Yakkala C Allen BD . Long-term neurocognitive benefits of FLASH radiotherapy driven by reduced reactive oxygen species. Proc Natl Acad Sci USA. (2019) 116:10943–51. doi: 10.1073/pnas.1901777116 31097580 PMC6561167

[23] Labarbe R Hotoiu L Barbier J Favaudon V . A physicochemical model of reaction kinetics supports peroxyl radical recombination as the main determinant of the FLASH effect. Radiother Oncol. (2020) 153:303–10. doi: 10.1016/j.radonc.2020.06.001 32534957

[24] Chabi S To THV Leavitt R Poglio S Jorge PG Jaccard M . Ultra-high-dose-rate FLASH and conventional-dose-rate irradiation differentially affect human acute lymphoblastic leukemia and normal hematopoiesis. Int J Radiat Oncol Biol Phys. (2021) 109:819–29. doi: 10.1016/j.ijrobp.2020.10.012 33075474

[25] Velalopoulou A Karagounis IV Cramer GM Kim MM Skoufos G Goia D . FLASH proton radiotherapy spares normal epithelial and mesenchymal tissues while preserving sarcoma response. Cancer Res. (2021) 81:4808–21. doi: 10.1158/0008-5472.CAN-21-1500 34321243 PMC8715480

[26] Bourhis J Sozzi WJ Jorge PG Gaide O Bailat C Duclos F . Treatment of a first patient with FLASH-radiotherapy. Radiother Oncol. (2019) 139:18–22. doi: 10.1016/j.radonc.2019.06.019 31303340

[27] Lin B Gao F Yang Y Wu D Zhang Y Feng G . FLASH radiotherapy: History and future. Front Oncol. (2021) 11:644400. doi: 10.3389/fonc.2021.644400 34113566 PMC8185194

[28] Patriarca A Fouillade C Auger M Martin F Pouzoulet F Nauraye C . Experimental set-up for FLASH proton irradiation of small animals using a clinical system. Int J Radiat Oncol Biol Phys. (2018) 102:619–26. doi: 10.1016/j.ijrobp.2018.06.403 30017793

[29] Mascia AE Daugherty EC Zhang Y Lee E Xiao Z Sertorio M . Proton FLASH radiotherapy for the treatment of symptomatic bone metastases: the FAST-01 nonrandomized trial. JAMA Oncol. (2023) 9:62–9. doi: 10.1001/jamaoncol.2022.5843 36273324 PMC9589460

[30] Daugherty EC Mascia A Zhang Y Lee E Xiao Z Sertorio M . FLASH radiotherapy for the treatment of symptomatic bone metastases (FAST-01): protocol for the first prospective feasibility study. JMIR Res Protoc. (2023) 12:e41812. doi: 10.2196/41812 36206189 PMC9893728

[31] van de Water S Safai S Schippers JM Weber DC Lomax AJ . Towards FLASH proton therapy: the impact of treatment planning and machine characteristics on achievable dose rates. Acta Oncol. (2019) 58:1463–9. doi: 10.1080/0284186X.2019.1627416 31241377

[32] Wei S Lin H Choi JI Simone CB Kang M . A novel proton pencil beam scanning FLASH RT delivery method enables optimal OAR sparing and ultra-high dose rate delivery: A comprehensive dosimetry study for lung tumors. Cancers (Basel). (2021) 13:5790. doi: 10.3390/cancers13225790 34830946 PMC8616118

[33] Kang M Wei S Choi JI Lin H Simone CB . A universal range shifter and range compensator can enable proton pencil beam scanning single-energy Bragg peak FLASH-RT treatment using current commercially available proton systems. Int J Radiat Oncol Biol Phys. (2022) 113:203–13. doi: 10.1016/j.ijrobp.2022.01.009 35101597

[34] Wieser HP Cisternas E Wahl N Ulrich S Stadler A Mescher H . Development of the open-source dose calculation and optimization toolkit matRad. Med Phys. (2017) 44:2556–68. doi: 10.1002/mp.12251 28370020

[35] Lin H Shi C Huang S Shen J Kang M Chen Q . Applications of various range shifters for proton pencil beam scanning radiotherapy. Radiat Oncol. (2021) 16:146. doi: 10.1186/s13014-021-01873-8 34362396 PMC8344212

[36] Folkerts MM Abel E Busold S Perez JR Krishnamurthi V Ling CC . A framework for defining FLASH dose rate for pencil beam scanning. Med Phys. (2020) 47:6396–404. doi: 10.1002/mp.14456 32910460 PMC7894358

[37] Combs SE Baumert BG Bendszus M Bozzao A Brada M Fariselli L . ESTRO ACROP guideline for target volume delineation of skull base tumors. Radiother Oncol. (2021) 156:80–94. doi: 10.1016/j.radonc.2020.11.014 33309848

[38] Kang M Wei S Choi JI Simone CB Lin H . Quantitative assessment of 3D dose rate for proton pencil beam scanning FLASH radiotherapy and its application for lung hypofractionation treatment planning. Cancers (Basel). (2021) 13:3549. doi: 10.3390/cancers13143549 34298762 PMC8303986

[39] Wei S Lin H Choi JI Press RH Lazarev S Kabarriti R . FLASH radiotherapy using single-energy proton PBS transmission beams for hypofractionation liver cancer: Dose and dose rate quantification. Front Oncol. (2021) 11:813063. doi: 10.3389/fonc.2021.813063 35096620 PMC8794777

[40] Wei S Lin H Isabelle Choi J Shi C Simone CB Kang M . Advanced pencil beam scanning Bragg peak FLASH-RT delivery technique can enhance lung cancer planning treatment outcomes compared to conventional multiple-energy proton PBS techniques. Radiother Oncol. (2022) 175:238–47. doi: 10.1016/j.radonc.2022.08.005 35961583

[41] Wei S Lin H Shi C Xiong W Chen CC Huang S . Use of single-energy proton pencil beam scanning Bragg peak for intensity-modulated proton therapy FLASH treatment planning in liver-hypofractionated radiation therapy. Med Phys. (2022) 49:6560–74. doi: 10.1002/mp.15894 35929404

[42] Cheng CY Xu LM Jing H Selvaraj B Lin HB Pennock M . The potential and challenges of proton FLASH in head and neck cancer reirradiation. Cancers (Basel). (2024) 16:3249. doi: 10.3390/cancers16193249 39409872 PMC11482542

[43] Chang CC Ma Y Kang M Paliwal B Morris Z Durkee B . Conditional sparing in FLASH radiotherapy: Conformal dosimetry and ALARA remain essential. J Appl Clin Med Phys. (2026) 27:e70664. doi: 10.1002/acm2.70664 42338090 PMC13291216

[44] Pan D Rong X Chen D Jiang J Ng WT Mai H . Mortality of early treatment for radiation-induced brain necrosis in head and neck cancer survivors: a multicentre, retrospective, registry-based cohort study. EClinicalMedicine. (2022) 52. doi: 10.1016/j.eclinm.2022.101618 36034411 PMC9399256

[45] Limoli CL Kramar EA Almeida A Petit B Grilj V Baulch JE . The sparing effect of FLASH-RT on synaptic plasticity is maintained in mice with standard fractionation. Radiother Oncol. (2023) 186:109767. doi: 10.1016/j.radonc.2023.109767 37385377 PMC11045040

